# Exploring the Immune Microenvironment in Early-Stage Mycosis Fungoides and Large-Plaque Parapsoriasis: Diagnostic and Prognostic Significance of CD47, CD163, and B7-H3

**DOI:** 10.3390/medicina62040678

**Published:** 2026-04-02

**Authors:** Rukiye Yasak Guner, Ramazan Oguz Yüceer, Ahmet Turan Unsal

**Affiliations:** 1 Department of Dermatology, Faculty of Medicine, Sivas Cumhuriyet University, 58140 Sivas, Turkey; ahmeturanunsal@gmail.com; 2 Department of Pathology, Faculty of Medicine, Sivas Cumhuriyet University, 58140 Sivas, Turkey; r.yuceer66@hotmail.com

**Keywords:** cutaneous lymphoma, mycosis fungoides, large-plaque parapsoriasis, tumor microenvironment, CD47, CD163, B7-H3, diagnostic biomarker

## Abstract

*Background and Objectives:* Mycosis fungoides (MF) is the predominant subtype of cutaneous T-cell lymphoma, whereas large plaque parapsoriasis (LPP) closely resembles early-stage MF, making differential diagnosis challenging. Immune markers, such as CD47, CD163, and B7-H3, play crucial roles in tumor immune evasion and macrophage polarization. However, their expression profiles and potential diagnostic or prognostic implications in early-stage MF and LPP remain poorly defined. Therefore, this study aimed to evaluate the expression of CD47, CD163, and B7-H3 in early-stage MF and LPP and analyze their associations with clinicopathological characteristics and patient outcomes. *Materials and Methods:* This retrospective study evaluated the immunohistochemical expression of CD47, CD163, and B7-H3 in 46 patients with early-stage mycosis fungoides (MF) and 46 patients with large plaque parapsoriasis (LPP). Expression levels were assessed using an immunoreactivity scoring system and analyzed for their associations with clinical parameters and disease-free survival (DFS). The study included patients diagnosed and followed at Sivas Cumhuriyet University between 1 March 2015 and 31 March 2025. *Results*: High CD47 expression was detected in 72.7% of MF patients, high B7-H3 expression in 45.7%, and high CD163 expression in 46.7% compared with LPP patients (*p* < 0.001). These markers showed positive correlations, and elevated expression, especially of B7-H3 and CD163, was associated with shorter disease-free survival in univariate analysis. *Conclusions*: The higher expression of CD47, CD163, and B7-H3 in early-stage MF compared with LPP suggests that these markers may contribute to the differential diagnosis and could represent potential therapeutic targets; however, their independent prognostic value requires confirmation in larger studies.

## 1. Introduction

Mycosis fungoides (MF), the most common type of cutaneous T-cell lymphoma, is characterized by the accumulation of CD4^+^ T cells in the skin. It usually presents as patches, plaques, and tumors and progresses slowly over several years [1]. Diagnosis can be challenging, particularly in the early stages, owing to its resemblance to inflammatory dermatoses, and it is often referred to as ‘the great imitator’ because of its ability to mimic a wide range of benign skin conditions [2].

Parapsoriasis is a chronic inflammatory skin condition that cannot be defined clinically or histomorphologically. It was first described by Brocq in 1902 and is now classified into two subtypes: small-plaque parapsoriasis (SPP) and large-plaque parapsoriasis (LPP). Although SPP is considered benign, progression to MF has been reported in 10–35% of LPP cases. The clinical, histological, and molecular similarities between LPP and early stage MF make it difficult to distinguish between them [3,4].

LPP has increasingly been regarded as a premalignant dermatosis with the potential to progress to Mycosis Fungoides. Several clinical, histopathological, and molecular studies have suggested that LPP may represent an early stage within the spectrum of cutaneous T-cell lymphoproliferative disorders, as a subset of patients gradually develop features diagnostic of MF during long-term follow-up. Consequently, careful clinical monitoring and repeated histopathologic evaluation are recommended in patients with LPP [5].

CD47, a membrane protein of the immunoglobulin superfamily, inhibits macrophage phagocytosis by interacting with SIRPα. Its overexpression in various solid tumors has been linked to immune evasion and poor prognosis, particularly in cutaneous squamous cell carcinoma [6,7]. Tumor-associated macrophages (TAMs) regulate immune responses in the tumor microenvironment; the CD68^+^ M1 phenotype exerts tumor-suppressive effects, whereas CD68^+^/CD163^+^ M2 macrophages promote progression and metastasis [8,9]. The immune checkpoint molecule B7-H3 (CD276) contributes to tumor progression by suppressing T cell activity and inducing M2 polarization. It also enhances aggressiveness through non-immune mechanisms, such as angiogenesis, invasion, metabolic reprogramming, and therapy resistance. Immunotherapeutic agents targeting B7-H3 are promising for multiple cancers [10,11,12].

This study aimed to evaluate the expression of CD47, CD163, and B7-H3 (CD276) in early-stage MF and LPP using histopathological methods and to investigate the relationship between these immune markers and clinicopathological characteristics. This study also aimed to investigate the potential of these immune markers as biomarkers for differential diagnosis and prediction of clinical prognosis in early-stage diseases.

## 2. Materials and Methods

This retrospective, cross-sectional study included patients aged ≥18 years diagnosed with early-stage mycosis MF or large-plaque LPP at Sivas Cumhuriyet University between 1 March 2015 and 31 March 2025. A total of 92 patients were enrolled, comprising 46 early-stage MF (patch/plaque) and 46 LPP, all with archived biopsy material available. All cases were reviewed again, and diagnoses were confirmed by dermatopathologists according to the International Society for Cutaneous Lymphomas (ISCL) and the European Organisation for Research and Treatment of Cancer (EORTC) criteria [13]. To minimize diagnostic circularity in the absence of T-cell receptor (TCR) gene rearrangement testing, explicit operational definitions were applied.

Early-stage MF was defined as (i) persistent or recurrent patches or thin plaques with a chronic course; (ii) at least one biopsy showing histopathological features consistent with MF, including epidermotropism of atypical lymphocytes with or without Pautrier microabscesses, basal alignment, and/or papillary dermal fibrosis; (iii) supportive immunophenotypic findings in the standard T-cell panel, such as aberrant antigen loss (particularly CD7 and/or CD5) and an appropriate CD4/CD8 profile; and (iv) clinicopathologic concordance [13].

**Figure 1:**
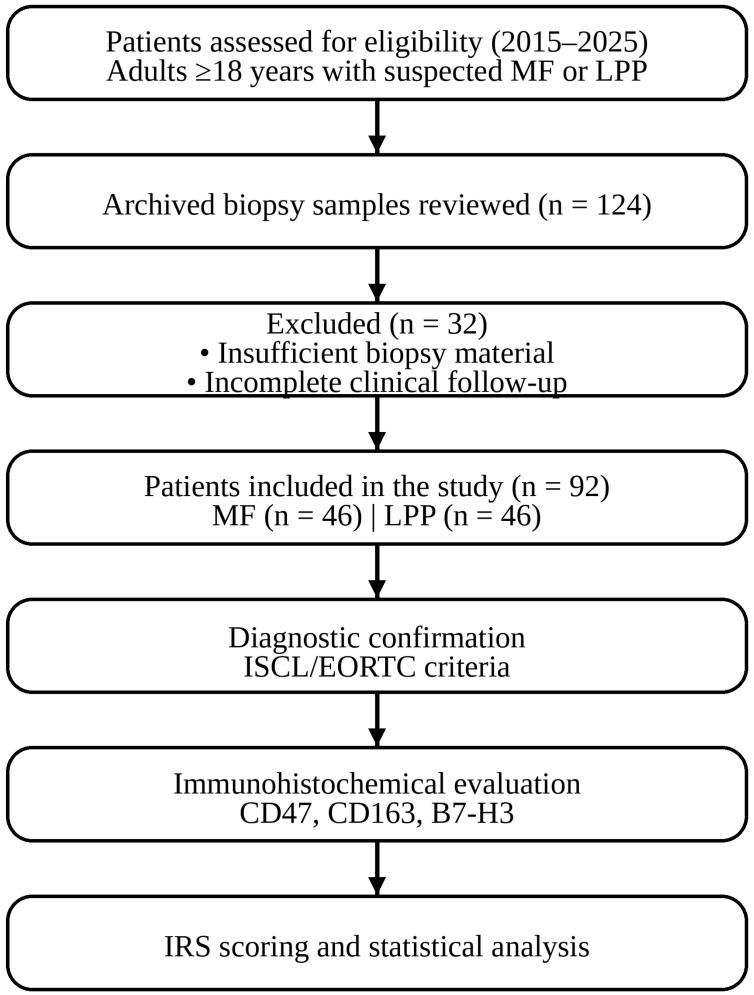
Patient selection flow diagram.

LPP was defined as (i) clinically typical large plaques; (ii) superficial perivascular lymphocytic infiltrates with limited or absent epidermotropism and without convincing cytologic atypia; (iii) absence of MF-type immunophenotypic aberrancy sufficient for MF diagnosis; and (iv) clinicopathologic concordance supporting a parapsoriasis-spectrum diagnosis. To address histopathological heterogeneity, ≥2 biopsies from distinct lesions were available for each patient. All specimens were reviewed, and most diagnostic biopsies were used for primary classification. Longitudinal clinical records were examined to provide evidence of diagnostic evolution. None of the LPP cases progressed to MF during the follow-up period. Patients with equivocal features underwent consensus re-review and were categorized according to the most conservative clinicopathologic interpretation. Immunohistochemistry was performed using a standard T cell panel (CD2, CD3, CD4, CD5, CD7, CD8, and CD30). Disease-free survival (DFS) was defined as the interval from the date of histopathologic diagnosis to the first documented evidence of disease progression or recurrence, including progression to a higher clinical stage or recurrence after complete or partial remission in early-stage mycosis fungoides, and progression to clinicopathologically confirmed mycosis fungoides or the development of new clinically and histologically progressive lesions in large-plaque parapsoriasis. Patients without documented progression or recurrence were censored at the date of the last follow-up. The median follow-up duration was 32.3 months for the MF group and 40.6 months for the LPP group. Staging was based on WHO–EORTC guidelines [14]. Clinical, laboratory, and immunohistochemical data are summarized in Figure 1.

### 2.1. Immunohistochemical Study of CD47-B7-H3-CD163

Hematoxylin and eosin (H&E)-stained slides from patients with MF and LPP were reevaluated. Formalin-fixed, paraffin-embedded (FFPE) tissue sections (4 µm thickness) were deparaffinized in xylene and rehydrated through graded alcohols. Antigen retrieval was performed by heat-induced epitope retrieval in citrate buffer (pH 6.0) in a pressure cooker for 20 min, followed by cooling at room temperature. Endogenous peroxidase activity was blocked using 3% hydrogen peroxide for 10 min.

The sections were then incubated overnight at 4 °C in a humidified chamber with the following primary antibodies: CD47 (rabbit monoclonal, clone EPR21794, 1:300 dilution; Abcam, Cambridge, UK), B7-H3 (rabbit monoclonal, clone EPR20115, 1:1000 dilution; Abcam, Cambridge, UK), and CD163 (mouse monoclonal, clone MRQ-26, 1:500 dilution; Cell Marque, Rocklin, CA, USA).

Immunodetection was performed using a polymer-based horseradish peroxidase (HRP) secondary detection system according to the manufacturer’s instructions. Diaminobenzidine (DAB) was used as the chromogen, and the sections were counterstained with Mayer’s hematoxylin, dehydrated, and mounted. Positive control tissues (placenta for CD47 and B7-H3 and tonsil for CD163) were processed in parallel. Staining was performed using an automated system.

A dermatopathology expert (R.O.Y.), blinded to the clinical data, independently evaluated all the slides. Immunoreactivity for CD47 and B7-H3 was defined as membranous and/or cytoplasmic staining, whereas CD163 expression was identified by cytoplasmic or granular staining of tumor-associated macrophages.

The immunoreactivity score (IRS) was calculated by combining the staining intensity and the proportion of positive cells. Staining intensity was graded on a 4-point scale (0, no staining, 1 = weak; 2 = moderate, 3 = strong), and the extent of staining was scored according to the percentage of positive cells (0 = 0%, 1 = <10%, 2 = 10–50%, 3 = >50%). IRS was obtained by multiplying the intensity and extent scores, yielding a total score ranging from 0 to 9. For statistical analysis, expression levels were categorized as negative/low (IRS 0–3) or high (IRS ≥ 4) [15,16].

### 2.2. Statistical Analysis

Statistical analyses were performed using SPSS version 27 (IBM Corp., Armonk, NY, USA). The normality of continuous variables was assessed using the Kolmogorov–Smirnov or Shapiro–Wilk test. Normally distributed variables are presented as mean ± standard deviation, whereas non-normally distributed variables are expressed as median (range). Categorical variables were compared using the chi-square test or Fisher’s exact test, as appropriate.

The required sample size was calculated using G*Power version 3.1.9.7. With an assumed effect size of 0.40, a type I error rate (α) of 0.05, and a statistical power of 95%, the minimum required sample size was determined to be 80 patient tissue samples. The final study cohort included 92 patients, exceeding the calculated minimum sample size.

DFS was estimated using the Kaplan–Meier method and compared using the log-rank test. Survival curves and number-at-risk tables were generated using software (version 4.4.3 R Foundation for Statistical Computing, Vienna, Austria) with the “survival” and “survminer” packages. Prognostic factors associated with DFS were evaluated using univariate Cox proportional hazards regression analysis. Variables that were statistically significant in the univariate analysis were included in the multivariate Cox regression model. A *p*-value < 0.05 was considered statistically significant.

## 3. Results

The study included 92 patients with a median age of 46 years (range, 19–91 years) and a slight female predominance (53.3%). Comorbidities and concomitant malignancy were present in 56.5% and 4.3% of patients, respectively. Table 1 summarizes the clinical and laboratory characteristics of the patients. Compared with LPP patients, MF patients were more frequently male, older, and had comorbidities (*p* < 0.05). The patch type was the most common clinical form (54.3%), and stage IA accounted for 58.7% of MF cases.

**CD47 expression:** High CD47 expression was observed in 72.7% of MF cases compared with 27.3% of LPP cases (*p* < 0.001). Within MF, high expression was most frequent in stage IA (47.3% vs. 2.7%, *p* < 0.001). Across the cohort, high CD47 expression was associated with age > 45 years (*p* = 0.005), male sex (*p* = 0.020), and comorbidity (*p* = 0.029). No significant associations were found with lesion site, concomitant malignancy, skin type, LDH, or β2-microglobulin, although patients with high expression levels more often had elevated LDH and β2-microglobulin levels.

**B7-H3 expression:** High B7-H3 expression was observed in 42 patients (45.7%) and was significantly associated with male sex (61.9%, *p* = 0.007), comorbidity (*p* = 0.022), MF diagnosis (90.5%, *p* < 0.001), and early stages (IA–IB, *p* < 0.001). No associations were observed between age, lesion site, concomitant malignancy, or laboratory parameters.

**Figure 2:**
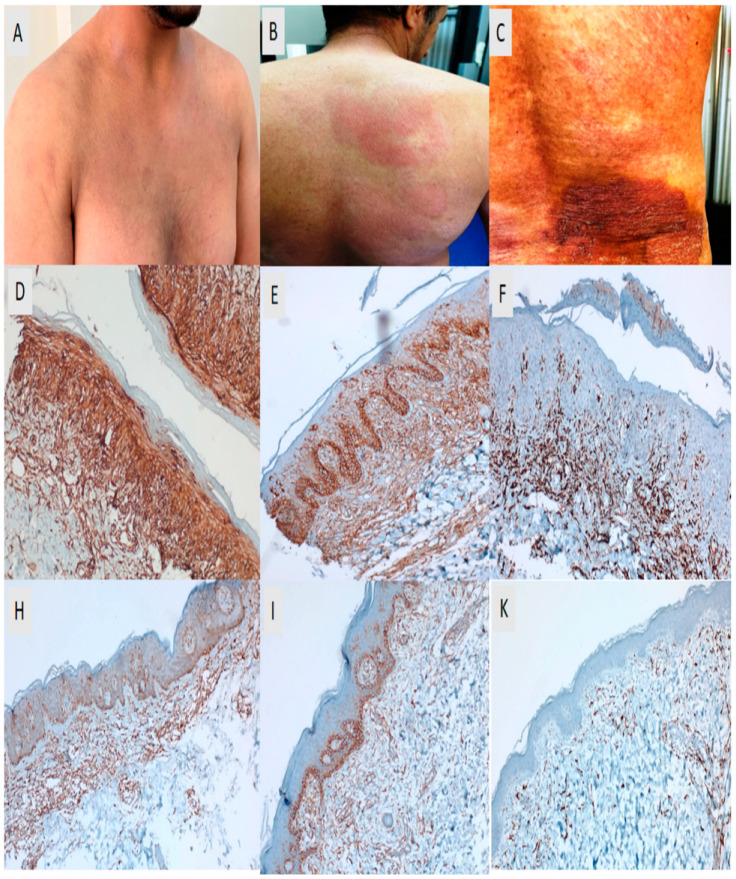
Clinical and immunohistochemical features of parapsoriasis and mycosis fungoides. Large plaque parapsoriasis is shown in panel (**A**), presenting with faint erythema and mild scaling on the anterior trunk. Patch-stage mycosis fungoides (MF) in panel (**B**) demonstrates localized, faintly erythematous patches with fine scaling on the back, while plaque-stage MF in panel (**C**) is characterized by a thickened, infiltrated, dark erythematous plaque with prominent scaling on the lumbar region. Panels (**D**–**F**) illustrate representative examples of high CD47, B7-H3, and CD163 expression, showing strong membranous and/or cytoplasmic staining in atypical lymphoid cells and diffuse cytoplasmic staining in dermal tumor-associated macrophages. Panels (**H**–**K**) present representative examples of low CD47, B7-H3, and CD163 expression, characterized by weak, focal, or limited staining patterns. All immunohistochemical images were obtained using the same magnification (DAB staining, ×200).

**CD163 expression:** High CD163 expression was detected in 43 patients (46.7%) and was significantly associated with comorbidity (69.8%, *p* = 0.014), MF diagnosis (86.0%, *p* < 0.001), and early-stage IA–IB (*p* < 0.001). No significant associations were identified with sex, age, lesion site, LDH, β2-microglobulin, or treatment modality (Table 2 and Figure 2).

### 3.1. Correlation Analysis

Moderate positive correlations were identified between CD47 and B7H3 (r = 0.485; *p* < 0.001), and between B7H3 and CD163 (r = 0.541; *p* < 0.001), whereas a weak positive correlation was observed between CD47 and CD163 (r = 0.368; *p* < 0.001). These findings suggested that these molecules act synergistically and contribute to a shared pathway in disease pathogenesis.

### 3.2. Survival Analysis

**Figure 3:**
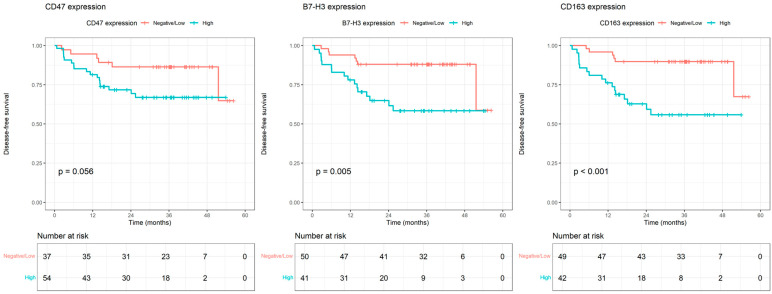
Kaplan–Meier curves for disease-free survival according to CD47, B7-H3, and CD163 expression.

During a median follow-up of 34.3 months, disease progression occurred in 24 patients (26.1%). The median Disease-Free Survival (DFS) was shorter in patients with high CD47 expression than in those with negative/low expression (39 vs. 49 months, *p* = 0.056). High B7-H3 expression was significantly associated with a shorter DFS (36 vs. 49 months, *p* = 0.005). Similarly, high CD163 expression was strongly associated with reduced DFS (35 vs. 50.6 months, *p* < 0.001) (Figure 3).

### 3.3. Univariate and Multivariate Cox Regression Analysis of Prognostic Factors

In the univariate Cox regression analysis, diagnosis, lesion type, stage, treatment type, and B7-H3 and CD163 expression were significantly associated with DFS (Table 3). MF was associated with a less favorable prognosis compared with LPP (*p* < 0.001), and plaque-type lesions predicted shorter survival than patch-type lesions (*p* = 0.001). Advanced disease stage and third-line treatment were also associated with poorer outcomes. At the molecular level, high B7-H3 (*p* = 0.008) and CD163 (*p* = 0.002) expression were associated with reduced survival, whereas CD47 showed borderline statistical significance (*p* = 0.064). Hazard ratios were calculated according to predefined reference categories.

In the multivariate Cox regression analysis, only lesion type remained an independent prognostic factor for DFS. Plaque-type lesions were significantly associated with shorter DFS compared with patch-type lesions (HR = 5.236, *p* = 0.009). Other variables, including stage, treatment type, diagnosis, and immune marker expression, did not retain statistical significance in the multivariate model (Table 3).

## 4. Discussion

The immunohistochemical findings of our study are highly consistent with the existing literature on the differential diagnosis of MF and LPP. However, these are unique in several respects. Notably, the significantly higher expression of CD163, CD47, and B7-H3 in MF cases than in LPP cases closely aligns with previous findings.

Kwantwi et al. emphasized that CD163^+^ macrophages are predominant in the tumour microenvironment (TME) of cutaneous T-cell lymphomas and play a role in supporting tumor progression [17]. El-Guindy et al. reported that CD163^+^ TAM density increases significantly with the transition from LPP to MF and that the CD163/CD68 ratio is lowest in LPP [18]. Furthermore, Mohammed et al. demonstrated that serum CD163 (sCD163) levels in MF patients were significantly higher than those in healthy individuals [19]. These systemic findings are consistent with the high tissue expression observed in our study, and further support its diagnostic value.

CD163^+^ TAMs are typically of the M2 phenotype, characterized by an immunosuppressive functional profile that supports tumor progression, promotes tissue remodelling, and enhances angiogenesis through VEGF and other pro-angiogenic mediators [20]. In our study, high CD163 expression was significantly more frequent in patients with MF (86.0%) than in those with LPP (*p* < 0.001), with high expression rates observed even in the early stages (stage 1A (46.5%) and Stage 1B (20.9%)). Importantly, survival analysis revealed that patients with high CD163 expression had a significantly shorter median disease-free survival (35.0 months, 95% CI: 28.1–41.9) compared to those with negative/low expression (50.6 months, 95% CI: 46.4–54.8; *p* < 0.001). This prognostic association is consistent with previous studies reporting that increased CD163^+^ TAM density correlates with worse clinical outcomes in MF [21].

Notably, our finding of high CD163 expression in MF, even at early stages, aligns with the evidence that M2 CD163^+^ TAMs can be therapeutically targeted, such as with brentuximab vedotin, to disrupt tumor-promoting functions and potentially improve clinical outcomes [22]. It is generally speculated that during early tumor development, monocytes have only recently migrated from circulation, the TME is not yet fully established, and macrophages are predominantly of the M1 phenotype with preserved antitumor activity [18]. However, our observation of high CD163^+^ TAM counts, even in the early MF stages, suggests that immunomodulatory cytokines, such as CCL22 and IL-10, may already drive M2 polarization at this stage, thereby suppressing local immune responses and initiating the formation of a tumor-supportive microenvironment.

In our study, we found that CD47 expression was significantly higher in MF than in LPP, which is consistent with the findings of Xiao and Akilov [23]. They reported that CD47 is highly expressed in MF and Sezary syndrome and is associated with a poor prognosis. The suppression of phagocytosis by CD47 through the ‘don’t eat me’ signal may play an important role in MF’s evasion of the immune system. Jiang et al. also demonstrated that anti-CD47 therapy reduced exhausted T cells in the immune microenvironment and increased the response of cytotoxic cells to MF [24]. Taken together, these data support the notion that high CD47 expression observed in our study may be diagnostically significant and represent a treatable target.

Our finding that B7-H3 (CD276) expression was significantly higher in MF than in LPP addresses a notable gap in the literature, as no previous studies have directly examined B7-H3 expression in MF. Although a review by Picarda et al. reported that B7-H3 is highly expressed in various solid tumors and associated with poor prognosis, it does not provide MF-specific data [25]. Given its recognized immunosuppressive effect on T cells, the high level of B7-H3 expression in a T-cell-derived lymphoma, such as MF, may represent a critical mechanism of immune evasion. The positive correlation observed between B7-H3 and CD163 expression (r = 0.541, *p* < 0.001) supports the role of B7-H3 in promoting M2 macrophage polarization, suggesting a synergistic pathway that fosters a tumor-permissive microenvironment and accelerates disease progression. Based on these findings, B7-H3 may serve as both a prognostic biomarker and potential immunotherapeutic target in MF. To our knowledge, this is one of the first studies highlighting its potential role in future targeted treatment strategies.

Although no significant differences were observed between patch and plaque lesions within the same stage, stage-wise analysis (1A, 1B, and 2A) revealed a significant stepwise increase in expression levels, indicating that upregulation occurred early in the disease course and remained consistent across lesion types within the same stage. Similarly, previous studies reported no significant differences in the expression of these markers between MF stages [18,19].

In our study, no statistically significant correlation was found between the expression levels of CD163, CD47, and B7-H3 and the laboratory parameters β2-microglobulin and LDH. Similarly, no significant relationship was observed between the expression levels of these markers and anatomical location or skin type. However, high CD47 expression was significantly associated with older age, male sex, and the presence of comorbidities; high B7-H3 expression was associated with male sex and comorbidity; and high CD163 expression was associated with comorbidity. These findings suggest that, even in early-stage disease, certain clinicodemographic factors may influence the tumor immune microenvironment, while systemic biomarkers such as LDH and β2-microglobulin may remain within normal limits at this stage. Although the association was not statistically significant, elevated LDH and β2-microglobulin levels were more frequently observed in patients with high CD47 expression; however, these findings should be interpreted with caution and do not indicate a confirmed relationship between CD47 expression and tumor burden. Given that both LDH and β2-microglobulin are recognized markers of disease activity and prognosis in haematological malignancies, this trend warrants further investigation in larger prospective cohorts [26].

The prognostic significance of B7-H3 and CD163 expression has been investigated in various malignancies, including urothelial bladder carcinoma, oral squamous cell carcinoma, head and neck squamous cell carcinoma, clear cell renal cell carcinoma, and other solid tumors [27,28,29,30,31]. In most of these studies, high B7-H3 expression was associated with poor prognosis; however, its status as an independent prognostic factor varied according to tumor type and analytical approach. Similarly, high CD163^+^ TAM infiltration has been linked to adverse outcomes in many solid tumors, although not universally across all cancer types.

In our MF cohort, B7-H3 expression was strongly correlated with CD163 expression (r = 0.541, *p* < 0.001), consistent with reports on bladder carcinoma [26]. Both markers were prognostic in univariate analysis but lost significance in multivariate models, likely reflecting modulation by other clinicopathological factors. Plaque-type lesions emerged as the only independent adverse prognostic factor that may account for the diminished impact of B7-H3 and CD163. These findings highlight the importance of evaluating immune checkpoint molecules and TAM markers within a broader tumour microenvironment.

This study demonstrated that CD47, B7-H3, and CD163 expression was more frequently observed in MF compared with LPP, even in early-stage disease. These findings suggest that these markers may contribute to the differential diagnosis between MF and inflammatory dermatoses. Considering their reported roles in immune regulation and macrophage-related pathways, these molecules may also represent potential targets for future therapeutic research.

This study had several limitations. The retrospective, single-center design may introduce selection bias and limit control over potential confounding factors. In addition, the inclusion of only early-stage disease restricts the generalizability of the findings to advanced-stage mycosis fungoides or to the broader spectrum of cutaneous T-cell lymphomas. The absence of T-cell receptor (TCR) gene rearrangement analysis represents another limitation, as molecular clonality testing can improve diagnostic precision, particularly in borderline cases within the parapsoriasis–mycosis fungoides continuum. Furthermore, immunohistochemistry itself has inherent limitations in terms of sensitivity and specificity and should always be interpreted in conjunction with standard histopathological evaluation. The reliance on immunohistochemical markers alone, without complementary molecular techniques, may therefore limit the diagnostic accuracy of the study.

Although standardized clinicopathologic criteria and consensus review were applied, the lack of molecular data may still reduce diagnostic resolution. Future prospective multicenter studies with larger cohorts, molecular profiling, and inclusion of all disease stages are required to validate these results and clarify the prognostic and potential therapeutic relevance of the investigated biomarkers.

## 5. Conclusions

CD47, CD163, and B7-H3 display distinct expression patterns in early-stage mycosis fungoides and large-plaque parapsoriasis and show meaningful associations with disease-free survival, supporting their potential diagnostic and prognostic value. Our results highlight the central role of the immune microenvironment in the early phases of cutaneous lymphomagenesis and indicate that immune checkpoint–related and macrophage-associated markers may be useful adjuncts in routine evaluation as well as promising candidates for future therapeutic exploration. Validation in larger, prospective cohorts is warranted.

## Figures and Tables

**Table 1:** Demographic and Clinicopathological Characteristics of Patients with Early-Stage Mycosis Fungoides and Large-plaque parapsoriasis.

Categorical Variables	n	%
**Age**		
**<45 years**	46	50.0%
**≥45 years**	46	50.0%
**Sex**		
**Male**	43	46.7%
**Female**	49	53.3%
**Comorbidity**		
**Absent**	40	43.5%
**Present**	52	56.5%
**Presence of Concurrent Malignancy**		
**Absent**	88	95.7%
**Present**	4	4.3%
**Localization**		
**Upper extremity**	73	79.3%
**Lower extremity**	19	20.7%
**Skin Type**		
**2**	2	2.2%
**3**	82	89.1%
**4**	8	8.7%
**LDH**		
**Normal**	42	91.3%
**Elevated**	4	8.7%
**β2-microglobulin**		
**Normal**	11	33.9%
**Elevated**	35	76.1%
**Tumor Type**		
**Patch**	25	54.3%
**Plaque**	21	45.7%
**Stage**		
**1a**	27	58.7%
**1b**	10	21.7%
**2a**	9	19.6%
**Treatment**		
**Topical therapy**	61	66.3%
**Phototherapy**	4	4.3%
**Systemic therapy**	27	29.3%

**Table 2:** Association Between CD47, B7-H3, and CD163 Expression and Clinicopathological Characteristics.

	CD47 Expression			B7-H3 Expression			CD163 Expression		
	Negative/Low n (%)	High n (%)	*p*	Negative/Low n (%)	High n (%)	*p*	Negative/Low n (%)	High n (%)	*p*
**Age**									
**<45**	25 (67.6)	21 (38.2)	0.005	29 (58.0)	17 (40.5)	0.071	28 (57.1)	18 (41.9)	0.105
**≥45**	12 (32.4)	34 (61.8)		21 (42.0)	25 (59.5)		21 (42.9)	25 (58.1)	
**Sex**									
**Male**	12 (32.4)	31 (56.4)	0.002	17 (34.0)	26 (61.9)	0.007	19 (38.8)	24 (55.8)	0.077
**Female**	25 (67.6)	24 (43.6)		33 (66.0)	16 (38.1)		30 (61.2)	19 (44.2)	
**Comorbidity**									
**No**	21 (56.8)	19 (34.5)	0.029	27 (54.0)	13 (31.0)	0.022	27 (55.1)	13 (30.2)	0.014
**Yes**	16 (43.2)	36 (65.5)		23 (46.0)	29 (69.0)		22 (44.9)	30 (69.8)	
**Presence of Concurrent Malignancy**									
**No**	36 (97.3)	52 (94.5)	0.469	48 (96.0)	40 (95.2)	0.623	47 (95.9)	41 (95.3)	0.641
**Yes**	1 (2.7)	3 (5.5)		2 (4.0)	2 (4.8)		2 (4.1)	2 (4.7)	
**Location**									
**Upper extremity**	27 (73.0)	46 (83.6)		38 (76.0)	35 (83.3)		40 (81.6)	33 (76.7)	
**Lower extremity**	10 (27.0)	9 (16.4)	0.164	12 (24.0)	7 (16.7)	0.273	9 (18.4)	10 (23.3)	0.374
**Skin type**									
**2**	1 (2.7)	1 (1.8)		1 (2.0)	1 (2.4)		2 (4.1)	0 (0.0)	
**3**	32 (86.5)	50 (90.9)	0.701	46 (92.0)	36 (85.7)	0.417	42 (85.7)	40 (93.0)	0.900
**4**	4 (10.8)	4 (7.3)		3 (6.0)	5 (11.9)		5 (10.2)	3 (7.0)	
**LDH**									
**Normal**	7 (100.0)	35 (89.7)	0.504	7 (87.5)	35 (92.1)	0.548	8 (88.9)	34 (91.9)	0.595
**Elevated**	0 (0.0)	4 (10.3)		1 (12.5)	3 (7.9)		1 (11.1)	3 (8.1)	
**β2-microglobulin**									
**Normal**	0 (0.0)	11 (28.2)	0.126	0 (0.0)	11 (28.9)	0.090	1 (11.1)	10 (27.0)	0.299
**Elevated**	7 (100.0)	28 (71.8)		8 (100.0)	27 (71.1)		8 (88.9)	27 (73.0)	
**Diagnosis**									
**MF**	6 (16.2)	40 (72.7)		8 (16.0)	38 (90.5)		9 (18.4)	37 (86.0)	
**LPP**	31 (83.8)	15 (27.3)	<0.001	42 (84.0)	4 (9.5)	<0.001	40 (81.6)	6 (14.0)	<0.001
**Tumor type**									
**Patch**	1 (16.7)	24 (60.0)		4 (50.0)	21 (55.3)		6 (66.7)	19 (51.4)	
**Plaque**	5 (83.3)	16 (40.0)	0.060	4 (50.0)	17 (44.7)	0.544	3 (33.3)	18 (48.6)	0.328
**Stage**									
**Stage 1a**	1 (16.7)	26 (65)		4 (50)	23 (60.5)		7 (71.4)	20 (54.1)	
**Stage 1b**	2 (33.3)	8 (20)	<0.001	0 (0.0)	10 (26.3)	<0.001	1 (14.3)	9 (24.3)	<0.001
**Stage 2a**	3 (50)	6 (15)		4 (50)	5 (13.2)		1 (14.3)	8 (21.6)	
**Treatment**									
**Topical therapy**	24 (64.9)	37 (67.3)		36 (72.0)	25 (59.5)		36 (73.5)	25 (58.1)	
**Phototherapy**	2 (5.4)	2 (3.6)	0.875	0 (0.0)	4 (9.5)	0.418	1 (2.0)	3 (7.0)	0.176
**Systemic therapy**	11 (29.7)	16 (29.1)		14 (28.0)	13 (31.0)		12 (24.5)	15 (34.9)	

LDH: Lactate dehydrogenase, MF: Mycosis Fungoides, LPP: large plaque parapsoriasis.

**Table 3:** Univariate and Multivariate Cox Regression Analysis for Disease-Free Survival.

Variable	Category	Univariate HR (95% CI)	*p*	Multivariate HR (95% CI)	*p*
**Age**	<45	Ref			
	≥45	0.90 (0.40–2.03)	0.796	–	–
**Sex**	Female	Ref			
	Male	0.54 (0.23–1.24)	0.146	–	–
**Comorbidity**	No	Ref			
	Yes	1.52 (0.64–3.60)	0.341	–	–
**Concurrent malignancy**	No	Ref			
	Yes	2.46 (0.57–10.57)	0.226	–	–
**Localization**	Upper extremity	Ref		Ref	
	Lower extremity	2.59 (1.09–6.19)	0.032	2.48 (0.61–10.02)	0.203
**Skin type**	Type 2	Ref			
	Type 3–4	1.93 (0.64–5.85)	0.246	–	–
**LDH**	Normal	Ref			
	Elevated	2.28 (0.66–7.86)	0.194	–	–
**β2-microglobulin**	Continuous	0.96 (0.86–1.06)	0.382	–	–
**Diagnosis**	LPP	Ref		Ref	
	MF	0.10 (0.03–0.35)	<0.001	0.20 (0.05–1.22)	0.602
**Tumor type**	Patch	Ref		Ref	
	Plaque	6.88 (2.28–20.72)	0.001	5.24 (1.52–18.09)	0.009
**Stage**	Stage 1a	Ref		Ref	
	Stage 1b–2a	0.17 (0.06–0.47)	0.001	1.10 (0.25–4.78)	0.904
**Treatment**	Topical	Ref		Ref	
	Phototherapy/Systemic	1.76 (1.16–2.68)	0.008	1.05 (0.56–1.99)	0.876
**CD47 expression**	Low	Ref		Ref	
	High	2.45 (0.95–6.30)	0.064	1.43 (0.36–5.69)	0.610
**B7-H3 expression**	Low	Ref		Ref	
	High	3.36 (1.38–8.20)	0.008	0.96 (0.24–3.95)	0.959
**CD163 expression**	Low	Ref		Ref	
	High	4.50 (1.76–11.52)	0.002	0.99 (0.26–3.76)	0.988

HR, hazard ratio; CI: Confidence Interval, LDH: Lactate dehydrogenase.

## Data Availability

The datasets used in this study can be made available by the corresponding author upon reasonable request, with permission from the Dermatology Department of Sivas Cumhuriyet University School of Medicine.

## Abbreviations

LPP	Large plaque parapsoriasis
MF	Mycosis fungoides
ISCL	International Society for Cutaneous Lymphomas
JAAD	Journal of the American Academy of Dermatology
LDH	Lactate dehydrogenase
SPP	Small plaque parapsoriasis
TAM	Tumour-associated macrophage
TCR	T-cell receptor
TME	Tumour microenvironment
WHO	World Health Organization
B7-H3	B7 homolog 3 (CD276)
H&E	Hematoxylin and eosin
DFS	Disease-free survival
